# 

**Published:** 1842-12-03

**Authors:** 


### MEDICAL EXAMINER,

EDITED BY

*J. B. BIDDLE, M. D. & W. W. GERHARD, M. D.*

VOL. I.] DECEMBER 3, 1842. [No. 49.

